# Yersinia Enterocolitica Sepsis in an Elderly Male With No Iron Overload: A Case Report From the Northeastern United States

**DOI:** 10.7759/cureus.26431

**Published:** 2022-06-29

**Authors:** Dina Alnabwani, Mehnoor Durrani, Ankita Prasad, Shashank Pandya, Kajal Ghodasara, Bassam I Hasan, Alexandra Greenberg, Pramil Cheriyath

**Affiliations:** 1 Internal Medicine, Hackensack Meridian Ocean University Medical Center, Brick, USA; 2 Internal medicine, Hackensack Meridian Ocean University Medical Center, Brick, USA; 3 Infectious Diseases, Hackensack Meridian Ocean University Medical Center, Brick, USA; 4 Gastroenterology, Hackensack Meridian Ocean University Medical Center, Brick, USA

**Keywords:** sepsis, iron overload, pork, food borne, cdc, bleeding, colitis, yersinia enterocolitica

## Abstract

*Yersinia enterocolitica (YE)* is a facultative anaerobic gram-negative coccobacillus of the genus *Yersinia* (the most common ones are *YE* serogroups *O:3; O:5,27; O:8;* and *O:9*). Its incubation period is typically 1-14 days. The symptoms of *YE *infection include fever, abdominal pain (which may mimic appendicitis), and diarrhea (which may be bloody and can persist for several weeks). It is most commonly reported in infants and children due to cross-contamination of their feeds and pacifiers by people handling pork products, especially while cooking chitterlings. Necrotizing enterocolitis has been described in infants following *YE* infections. Adults who are immunocompromised or in an iron-overload state can develop sepsis with *YE* infection, which has a high fatality rate. Post-infectious sequelae like reactive arthritis and erythema nodosum can occur in certain HLA types. The diagnosis is made by isolating the organism from the body fluids, stool. The gastrointestinal (GI) pathogen panel by polymerase chain reaction* *(PCR) is helpful in making an early diagnosis. In this report, we discuss a case of an elderly male from a nursing facility who presented with abdominal pain, vomiting, GI bleeding, and sepsis. He required a brief ICU stay and pressor support. GI pathogen panel was instrumental in the early diagnosis of *YE*. This condition is not often reported in the Northeastern US. Using GI pathogen PCR testing will lead to the detection of more cases of *YE* in geographical regions where it was not considered prevalent.

## Introduction

*Yersinia enterocolitica* (*YE*) is a facultative anaerobic gram-negative coccobacilli (*YE* serogroups: *O:3; O:5,27; O:8;* and *O:9)* [1]. Its incubation period ranges from one to 14 days. *YE* infection is a zoonotic disease that causes gastroenteritis through the contamination of food and water by contact with infected pigs, cattle, rabbits, rodents, dogs, cats, and sheep [1]. Most infections are caused by consuming raw or undercooked pork, unpasteurized milk, and contaminated water [1-2]. The Centers for Disease Control and Prevention (CDC) monitors the frequency of *YE* infections. Since 2014, the incidence of yersiniosis has been less than 0.30 per 100,000 people [3]. *YE* gastroenteritis predominantly affects children and infants and causes fever, abdominal pain, and bloody or mucoid diarrhea. Necrotizing enterocolitis can occur in infants. Reactive arthritis affecting the wrists, knees, and ankles, and erythema nodosum can also occur. People with HLA-B27 are more susceptible to reactive arthritis. It causes pseudoappendicitis, mesenteric lymphadenitis, skin rashes, joint pain, and endocarditis in adults. Lymphoid tissues and Peyer patches get colonized after a gastrointestinal (GI) infection, and the bacterial shedding in the stool can continue up to three months after clinical improvement [1]. Hence, its detection is vital to contain the infection spread. The diagnosis is made by isolating the organism from stool or other body fluids. Most infections are usually self-limiting, and antibiotics are prescribed for severe conditions and immunocompromised or elderly individuals. Antimicrobial therapy does not affect post-infectious sequelae. Sepsis is seen in immunocompromised adults or an iron-overload state like hemochromatosis and thalassemia with an overall fatality rate of 50% [4]. If *YE* infection is suspected, the clinical laboratory should be notified to culture on cefsulodin-Irgasan-novobiocin* *(CIN) agar media, which is specific. We report a case of an elderly male from a nursing facility who presented with sepsis following abdominal pain, vomiting, and GI bleeding.

## Case presentation

An 88-year-old-male presented to the emergency room (ER) from a skilled nursing facility in the Northeastern US for the evaluation of sudden onset of fever, emesis, abdominal pain, and one episode of bloody diarrhea. He complained of feeling dizzy and weak and had a non-itchy blotchy rash on his bilateral lower limbs. He had no chills, jaundice, weight loss, anorexia, chest pain, or palpitations. He did not have any past history of GI bleed. However, he had an extensive medical history of coronary artery disease, atrial fibrillation, aortic valve replacement, diabetes mellitus, hypertension, hypothyroidism, dementia, and polyneuropathy. He was on multiple medications, including aspirin and apixaban. He had been living at the care facility since his spouse passed away. He was drowsy and confused at the time of presentation but oriented to time, place, and people. His temperature was 98.5°F, heart rate was 84/minute, blood pressure was 90/54 mmHg, and SpO_2_ was 99% on room air. He had mottling of the skin on the legs bilaterally, 2+ pitting edema of the feet, and no jaundice was present. The abdomen on examination was soft with no distension or organomegaly. Mild abdominal tenderness was observed in the left lower abdomen and a dark red stool was present in the diaper. All other systemic examinations were normal.

**Figure 1:**
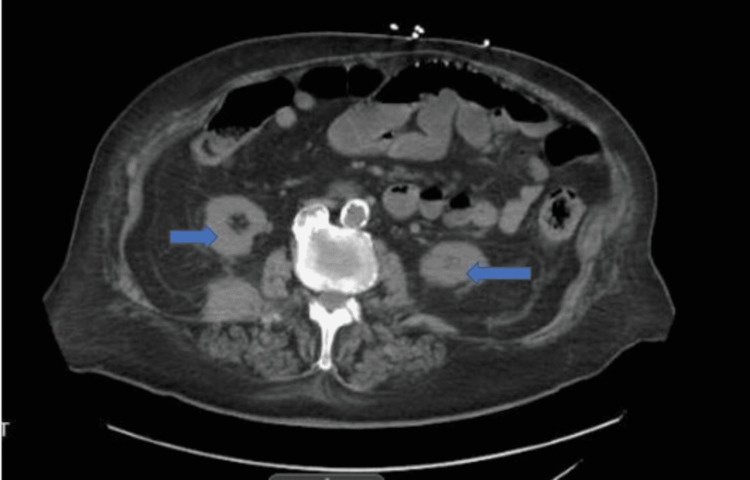
CECT of the abdomen (transverse section) with thickened intestines suggestive of colitis (arrows) CECT: contrast-enhanced computed tomography

**Figure 2:**
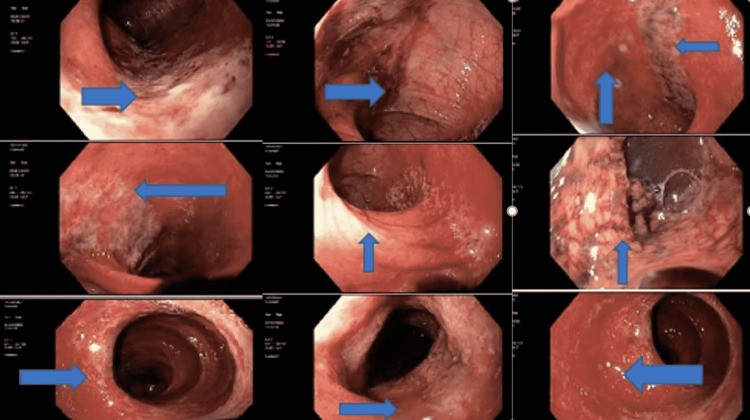
Sigmoidoscopy images Blue arrows show edematous friable mucosa with ulceration and submucosal edema suggestive of colitis

He received intravenous (IV) normal saline boluses and was then started on IV fluids: IV ampicillin/sulbactam because of sepsis, and IV pantoprazole for GI bleed. Apixaban and aspirin were withheld because of bleeding. He required pressor support and ICU admission briefly. He had leucocytosis (20,800/mm^3)^ with prominent neutrophilia (absolute neutrophil count: 18,200 cells/mm^3^). He had a hemoglobin level of 15.3 gm/dl, C-reactive protein (CRP) of 36 mg/L, and prothrombin time/international normalized ratio (PT/INR) was 23/2.04. The stool was negative for *Clostridium difficle (CD)*; blood, urine, stool cultures, and a GI pathogen panel (PCR) were also sent. He had mildly raised blood urea (47 mg/dl) and creatinine (2.42 mmol/dl), which was attributed to pre-renal azotemia, and his mildly increased troponin (0.07 ng/ml) was attributed to demand ischemia. Urine culture was positive for *Enterococcus faecalis*; a contrast-enhanced CT (CECT) of the abdomen suggested mild wall thickening in the left colon, indicative of early or mild colitis (Figure 1). Gastroduodenoscopy and flexible sigmoidoscopy were done, and they showed two clean-based antral ulcers without bleeding. There was circumferential inflammation of the right colon with ulcerated and friable mucosa, raising concerns for possible Inflammatory bowel disease vs. ischemia, as shown in Figure 2.

GI pathogen panel was positive for *YE. *The patient was started on IV doxycycline. Gastroenteritis was not reported in any of the other residents of the care facility. We ruled out any iron-overload state, and he had not received any blood transfusion recently. So we attributed the sepsis associated with *YE* to his immunocompromised state due to old age and multiple comorbidities. His condition gradually improved and he was later discharged in a stable condition.

## Discussion

In 2012, CDC set up FoodNet, an active surveillance network for foodborne infections [3]. CDC monitors the frequency of *YE* infections. The incidence of yersiniosis in FoodNet sites in 2014 was 0.28 cases per 100,000 population. This met the US Healthy People 2020 target for yersiniosis of 0.30 or fewer cases per 100,000 [3].*YE* cases have been mostly reported from specific regions in the western US and not traditionally from the Northeastern US [5] where our patient hails from.

Genus*Yersinia* comprises three major pathogens: *YE, Y. pseudotuberculosis, *and* Y. pestis*. *YE* is a foodborne pathogen, and humans are the incidental host. Pigs are the most important reservoir of the strains that are pathogenic in humans, i.e., serogroups* O:3, O:5,27, O:8, *and* O:9; *and bio groups* 1B, 2, 3, *and* 4 *[1,6]. In the US, there is a strong association between the condition and the consumption of undercooked “chitterlings” [3]. Outbreaks have also been caused by unpasteurized milk, tofu, greens, vegetables, and many other food products [7]. *YE *is a cold-tolerant bacteria that can continue to grow and multiply at low temperatures in refrigerated foods and stored blood. Increased incidence of *YE* infection is seen in colder months, especially around Christmas in temperate regions.

*YE* is an intracellular pathogen and survives in macrophages and lymphoid tissue. Adherence, invasion, and survival in lymphoid tissue depend on various virulence factors like invasin, attachment-invasion locus, adhesin A, outer membrane proteins that prevent opsonization, and phagocytosis by blocking the secretion of TNF-alpha and IL-8. It also produces a heat-stable enterotoxin that causes diarrheal disease [8]. It has ureases to survive passage through the acid environment of the stomach. Changes in host conditions and environment can result in gene expression and subsequent virulence changes. *Yersinia* cannot chelate iron, and it is an essential growth factor [8]. The association between *YE* septicemia and individuals with iron overload or those receiving iron-chelating therapy is fascinating. An iron-rich atmosphere is required for *YE* to survive.

*YE* infections commonly present with diarrhea, blood or mucus in stool, tenesmus, low-grade fever, abdominal pain, nausea, and vomiting. Children are mostly infected with *YE *serotype* O:3,* whereas adults are most commonly infected with serotype* O:9. *Pharyngitis can be present along with gastroenteritis in adults. It causes infections mostly in infants and young children. Infections in them are associated with cross-contamination of their food while handling pork products. It can mimic appendicitis with fever, tenderness, and right lower abdominal pain with leukocytosis in teenagers and young adults. Symptoms of pseudoappendicitis have often led to unnecessary appendectomies. In one study from Ireland, it was estimated (based on serologic testing) that up to 4% of patients undergoing appendectomies had acute *YE* infections [9]. At the time of surgery, such patients tend to have enlarged mesenteric lymph nodes (mesenteric adenitis) with a normal or slightly inflamed appendix [9]. *YE* may be cultured from involved lymph nodes and the distal ileum [9]. The risk of invasive disease increases in persons with chronic illnesses or immunosuppressive diseases, conditions that predispose to iron overload like thalassemia and hemochromatosis, therapy with deferoxamine, and transfusion-associated infections; these patients can present with septic shock in the setting of *YE *infections. The fatality rate among persons with transfusion-associated sepsis is high. It can cause extraintestinal manifestations like pharyngitis, soft tissue infection, osteoarticular infections, myocarditis, glomerulonephritis, and suppurative lymphadenitis. Post-infectious sequelae are caused by cross-reactivity with the bacterial antigen, mostly in HLA-B27-positive persons. Reactive arthritis due to *YE* tends to affect multiple larger joints. Joint symptoms appear 7-14 days after the GI symptoms and last for up to four months. Erythema nodosum lesions appear 2-14 days after the GI symptoms. These are more common in adult females and resolve on their own.

Colonization of lymphoid tissues in the intestines and shedding of the bacteria continue to occur up to 120 days after the resolution of symptoms. Hence, its detection is vital to contain the spread of infection. A high index of suspicion is maintained in cases of gastroenteritis with or without blood and mucus in stool and sepsis in persons with iron-overload states, and adult patients with diarrhea associated with pharyngitis or diarrhea with left lower abdomen tenderness. It needs CIN media for culture and isolation, and the laboratory should be notified if this infection is suspected. Commercial tests like the GI pathogen panel tests for *Campylobacter*, *Salmonella*, *Shigella*, *Vibrio*, *YE*, Shiga toxin 1 and 2, norovirus, and rotavirus can now identify multiple enteric pathogens, including *YE, *using genetic/molecular approaches. Using these tests can increase the detection rate of *YE* in suspected infections.

Fluoroquinolones are the drugs of choice for *YE* infections; third-generation cephalosporins, trimethoprim-sulfamethoxazole, doxycycline, and aminoglycosides are also effective [10]. Penicillin, imipenem, and first- and second-generation cephalosporins are ineffective and should not be used for treatment [10]. Antimotility agents are contraindicated because of the increased risk of severe infection. Hand-washing and control of environmental cross-contamination are principal measures in reducing the spread of enteric pathogens in daycare centers, healthcare settings, pet-care facilities, and households. Enteric precautions should be instituted for the care of patients who have been hospitalized with an infection.

## Conclusions

*YE *can cause infection across all age groups and is widely prevalent in the US. Diseases in the elderly, immunocompromised, and persons with iron-overload states like thalassemia and hemochromatosis are prone to sepsis with *YE* with very high fatality rates. Commercial tests like GI pathogen panels can help identify the infection early on and help identify many more cases than previously thought to have existed. It is crucial to identify this pathogen for controlling the spread of infection because bacterial shedding can continue for up to three to four months after the resolution of illness. Hand-washing and food hygiene are important to prevent this infection.
